# Sirtuin 3 deficiency promotes acute kidney injury induced by sepsis *via* mitochondrial dysfunction and apoptosis

**DOI:** 10.22038/ijbms.2021.54905.12312

**Published:** 2021-05

**Authors:** Heng Fan, Jian-wei Le, Min Sun, Jian-hua Zhu

**Affiliations:** 1 Department of Intensive Care Unit, Ningbo First Hospital, Ningbo, Zhejiang Province, P.R China

**Keywords:** Acute kidney injury, Apoptosis, Mitochondria, Sepsis, Sirtuin3

## Abstract

**Objective(s):**

To explore the regulation mechanism of Sirtuin 3 (SIRT3) on the mitochondrial function and apoptosis of acute kidney injury (AKI) in septic mice.

**Materials and Methods:**

The sepsis-induced AKI model was constructed in the wild-type and SIRT3 knockout (KO) mice, and the levels of serum creatinine (Scr) and plasma kidney injury molecule 1 (pKIM-1) in mice were detected by ELISA. The mitochondrial damage of kidney tubular epithelial cells (KTEC) was observed by electron microscopy, the apoptosis of KTEC was detected by TUNEL assay, and the mRNA levels of SIRT3, Bax, Caspase-3, and Bcl-2 were detected by RT-qPCR.

**Results:**

SIRT3 KO caused increased expression of Scr, pKIM-1, and inducible nitric oxide synthase protein in the kidneys of septic mice, and decreased the levels of superoxide dismutase, catalase, and mitochondrial complex enzymes I/II/III/IV. SIRT3 deficiency exacerbated histopathological and mitochondrial damage to the proximal tubules of the kidney. In addition, SIRT3 KO resulted in a significantly increased apoptosis of KTEC, increased the mRNA levels of Bax and Caspase-3, and decreased the mRNA levels of Bcl-2.

**Conclusion:**

Our study suggests that SIRT3 deficiency promotes sepsis-induced AKI *via* increasing oxidative stress, mitochondrial dysfunction, and inducing apoptosis.

## Introduction

Acute kidney injury (AKI) is characterized by a sudden decrease in glomerular filtration rate, which can be caused by various causes such as renal ischemia, nephrotoxic drugs, sepsis, and urinary tract obstruction (1). The main clinical manifestations of AKI are increased serum creatinine (Scr) concentration and oliguria, the treatment measures include symptomatic treatment for the cause, protection of kidney function, prevention and management of complications, etc (2). The studies indicated that the incidence of AKI in patients with sepsis is 35–50%, and the mortality rate is as high as 35%, and the 90-day mortality rate increases with the severity of the disease (3, 4). Some surviving patients will inevitably develop end-stage kidney disease, threatening the life and health of all human beings.

Mitochondrial injury is one of the pathophysiologies of AKI, and silent signal pathways of mitochondrial protein homeostasis play an important role in mitochondrial production, division, fusion, and apoptosis (5). Sirtuins (SIRTs) are composed of approximately 270 amino acid residues, and which have two different domains and a crack between the domains, containing the binding site of nicotinamide adenine dinucleotide, binding nicotinamide adenine dinucleotide after a catalytic reaction (6). Studies found that the SIRT family has seven subtypes, and the difference in N-terminal and C-terminal structures determines the different positioning and function of each subtype in each subcellular structure: SIRT1, SIRT6, and SIRT7 exist in the nucleus, SIRT2 is found in the cytoplasm and nucleus, while SIRT3, SIRT4, and SIRT5 are mainly found in the mitochondria (7, 8). SIRT3, SIRT4, and SIRT5 are responsible for the regulation of protein acetylation (9). Therefore, SIRT3 may have potential value in the mechanism of the occurrence and development of AKI. 

In the present study, SIRT3 knockout (KO) mice were used to construct a sepsis model. The present study aims to further study the regulation mechanism of SIRT3 on the mitochondrial function and apoptosis of kidney tubular epithelial cells (KTEC) in septic mice and to provide a new therapeutic target for AKI induced by sepsis.

## Materials and Methods

***Animals and grouping***

SIRT3 KO C57BL6 mice were purchased from Jackson Laboratory (Farmington, Connecticut, USA), male, 6–8 weeks old. Wild-type (WT) C57BL6 mice were purchased from the Experimental Animal Center of Zhejiang University (Hangzhou, China). We kept all mice in a standard environment, keeping light and darkness for 12 hr each, during which the mice were free to eat and drink. The mice were randomly divided into 4 groups (n=10): WT-Sham group, KO-Sham group, WT-cecal ligation and perforation (CLP) group, and the KO-CLP group. According to a previous protocol, we used the CLP method to construct a model of sepsis (10). In short, we anesthetized mice with 1.5% pentobarbital (0.1 ml/20 g) intraperitoneally, cut the abdominal wall at the midline, ligated the distal cecum, and pierced the proximal cecum with a 27 gauge needle and extruded a small amount of feces, sutured the incision after returning to the cecum. The procedures of the Sham group were the same as that of the CLP group, except for ligation and perforation. At 24 hr after CLP, the mice were sacrificed by neck fracture, and the blood and kidneys were taken for subsequent experiments. All experimental protocols involved in this study were approved by the Experimental Animal Ethics Committee of Ningbo University, and all steps were carried out in accordance with the experimental protocols and standard procedures.

***H*** ***&*** ***E staining***

We fixed part of the right kidney in 4% paraformaldehyde for 24 hr, washed twice with PBS, and embedded in paraffin after dehydration of conventional tissue. We used a microtome to cut the paraffin block into 2–3 μm sections, and performed H&E staining after routine deparaffinization and removal, and observed the pathological damage under a common optical microscope. Quantitative scores of kidney tubular epithelial histopathological damage: normal, 0; 0-25%, 1; 25-50%, 2; 50–75%, 3; >75%, 4. Specific criteria: kidney tubular shedding and atrophy, cell edema and rupture, basement membrane exfoliation, interstitial inflammatory cell infiltration, and vacuolation (11). 

***Immunochemistry***

We cut the paraffin block into 2–3 μm sections, after dehydration and clearing, the slices were put in the incubator for 30 min, then taken out and cooled for 15 min. We added the primary antibody [SIRT3 (R&D Co., Shanghai, China) or inducible nitric oxide synthase (iNOS, R&D Co., Shanghai, China)] according to the instructions, counterstained with the ABC kit, and observed under an optical microscope.

***Transmission electron microscopy***

We fixed the kidney cortex with 4% paraformaldehyde and 0.02% picric acid and fixed it again with 1% tetraoxide and 1.5% potassium ferrocyanide. We dehydrated the treated kidney and embedded it in resin, and used Accu-Edge to cut the kidney into ultra-thin sections. After staining the sections with lead citrate, we observed the sections on a JEM 1400 electron microscope (Automation Technology Co., Ltd., Jieou, Japan) and took pictures with a Veleta 2K digital camera (Daten Group Co., Ltd., Shanghai, China). 

***TUNEL assay***

We used the TUNEL method (R&D Co., Shanghai, China) to measure the apoptosis of KTEC. Specific steps: cut the paraffin-embedded slices into thick 2-3 um slices, routinely dehydrated and transparent, and rinsed twice with PBS. The antibody was added according to the manufacturer’s instructions, observed under an ordinary microscope, photographed with a digital camera, and TUNEL positive cell nuclei were calculated.

***Enzyme-linked immunosorbent assay (ELISA)***

We used the ELISA kit to measure the levels of Scr (Baichuan Co. Ltd., Ningbo, China) and plasma kidney injury molecule-1 (pKIM-1) (Baichuan Co. Ltd., Ningbo, China), and all experimental protocols were strictly in accordance with the instructions. 

***RT-qPCR***

We used the RNeasy mini kit (Thermo Fisher Scientific, Beijing, China) to extract total RNA from the kidney cortex, and followed the procedure for RNA purity identification. We used the following mRNA primers: SIRT3, 3’-AAA GCA ACT ACC ACG AGC CAC CAG CAG CGC-5’, 5’-CGC CAG CGT CGT CCA CTC TGC CTT CTA CAC-3’; Bcl-2, 3’-ACG CGA CGG CGA CGA CCC CAG CTC ACC-5’, 5’-ACG CGG CGA GCA CCA CGC CAA CA-3’; Bax, 3’-CAG CTC CTT CAT TCA ACG CAA CAT CCC CA-5’, 5’- CAC CAA CAG GAC CCC CAT CTT CAG GCC CA-3’; Caspase-3, 3’-AGG ACT CTT CTA ACG GAA GAT TAC CCA CTA CAA-5’, 5’- ACG GTA CTA CCC CTA AAT ACG CGT CA-3’. The above primers were provided by Huada Gene Company (Shanghai, China). The reaction conditions were set: denaturation at 95 °C, rapid cooling to 40–60 °C, primer annealing at 65 °C, and extension of the primer chain along the template under the action of Taq DNA polymerase for a total of 35 cycles.

***Anti-oxidant enzyme detection***

The levels of superoxide dismutase (SOD) (Abcam Co., Beijing, China) and catalase (CAT) activity (Solarbio Co., Beijing, China) were detected according to the manufacturer’s instructions. The fresh kidney tissue (0.2 g) was taken, ground on ice, centrifuged at 15000 r/min for 15 min, and the supernatant was taken for determination of antioxidant enzymes. A reaction system with 3 ml (0.3% H_2_O_2_ 1 ml, H_2_O 1.95 ml) was added, and 0.05 ml enzyme solution was added to start the reaction system. The OD reduction rate at a wavelength of 240 nm was measured, and SOD and CAT activities were calculated according to the rate of change of absorbance.

***Mitochondrial complex enzyme detection***

We took the kidney cortex, routinely homogenized on ice, centrifuged, added a disposable mitochondrial extraction reagent, homogenized, centrifuged, removed the supernatant, added lysed enzymes, mixed it, and allowed it to stand on ice for 15 min. We used spectrophotometry to measure the activity of mitochondrial enzyme complexes.

***Statistical analysis***

All data were expressed as mean±SEM or percentage. We used Prism 6.02 for mapping, the one-way ANOVA method for comparison between multiple groups, and Tukey’s *post hoc* test. We set *P*<0.05 to be considered statistically significant.

## Results

***The protective effect of SIRT3 on CLP-induced AKI***

**Figure 1:**
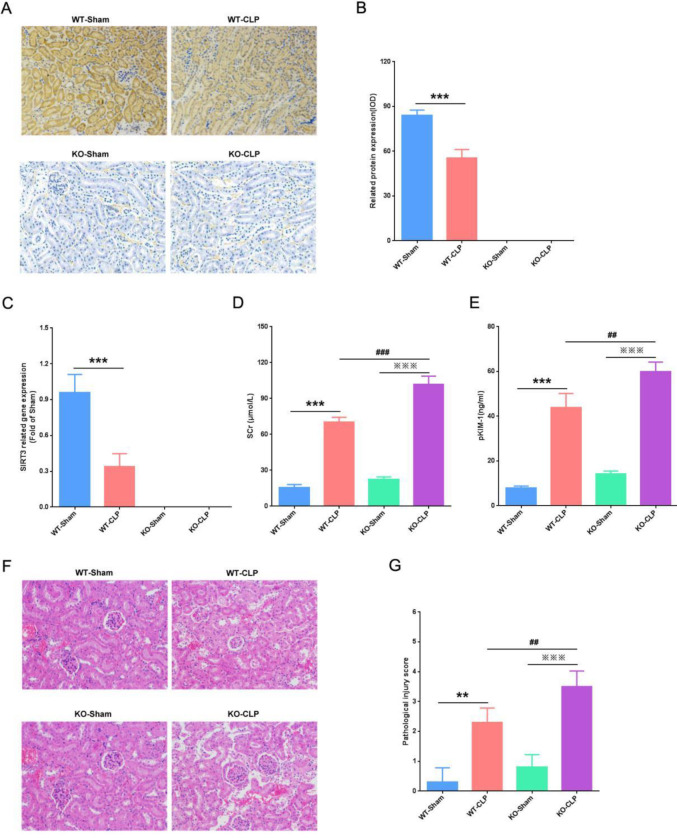
Protective effect of SIRT3 on CLP-induced AKI. (A) Immunohistochemical detection of SIRT3 protein expression in the kidney (IHC, ×200). (B) Quantitative analysis of SIRT3 protein expression. (C) Expression of SIRT3 mRNA in the kidney. (D) Expression levels of Scr in each mice group. (E) Expression levels of pKIM-1 in each mice group. (F) Pathological damage of kidney tissue (H&E, ×200). (G) Pathological score of kidney tissue. SIRT3, Sirtuin3; CLP, cecal ligation, and perforation; AKI, acute kidney injury; Scr, serum creatinine; pKIM-1, plasma kidney injury molecule-1. Compared with WT-Sham group, ^**^P<0.01, ^***^P<0.001; compared with KO-Sham group, ^***^P<0.001; compared with WT-CLP group, ^##^*P*<0.01, ^###^*P*<0.001

To confirm the protective effect of SIRT3 on CLP-induced AKI, we detected the protein and mRNA expression of SIRT3 in kidneys. Our immunohistochemistry results showed that SIRT3 protein was highly expressed in the kidneys of the WT-Sham group, and CLP caused SIRT3 protein to be significantly reduced (Figures 1A and B). Consistent with the above results, our RT-qPCR results showed that the mRNA level of SIRT3 was high in the WT-Sham group, compared with that, the mRNA level of SIRT3 was significantly decreased in the WT-CLP group (Figure 1C). Furthermore, we used the ELISA method to detect the levels of Scr and pKIM-1. We found that compared with the WT-Sham group, CLP caused Scr and pKIM-1 levels to significantly increase, and these two indicators were more significantly elevated in KO-CLP mice (Figures 1D and E). The above results preliminarily confirmed the protective effect of SIRT3 on septic AKI, and our pathological analysis results also confirmed this. We found that in the WT-CLP group, KTEC were shed, the glomerular capsule was ruptured, and the renal interstitium was scattered in the inflammatory cells. In the KO-CLP group, KTEC were extensive and the glomeruli were completely destroyed, and a large number of inflammatory cells filled in the interstitium (Figure 1F). Compared with the WT-CLP group, the kidney tissue injury score of the KO-CLP group was significantly increased (Figure 1G). Our results suggested that SIRT3 had a protective effect on CLP-induced AKI. 

***The effect of SIRT3 on CLP-induced oxidative stress***

**Figure 2:**
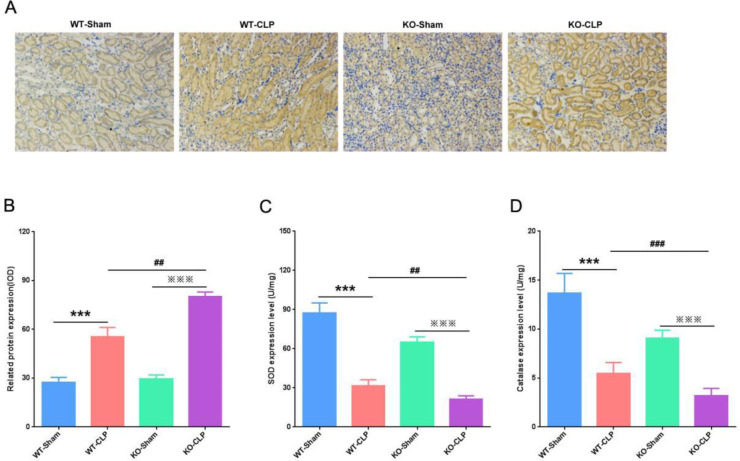
SIRT3 activates the activity of antioxidant enzymes in the kidneys. (A) Immunohistochemical detection of iNOS protein expression in the kidney (×400). (B) Quantitative analysis of iNOS protein expression. (C) Expression levels of SOD in the kidney. (D) Expression level of CAT in the kidney. SIRT3, Sirtuin3; iNOS, inducible nitric oxide synthase; SOD, superoxide dismutase; CAT, catalase. Compared with WT-Sham group, ***P<0.001; compared with KO-Sham group, *P*<0.001; compared with WT-CLP group^, ##^*P*<0.01, ^###^*P*<0.001

As an oxidase inducer, iNOS can activate and up-regulate various oxidases to inhibit oxidative stress(12). To investigate the effect of SIRT3 on oxidative stress during sepsis-induced AKI, we used immunohistochemistry to detect iNOS protein expression. We found that iNOS protein was both expressed at low levels in WT-Sham and KO-Sham groups, and CLP caused the expression of iNOS protein to significantly increase. Moreover, compared with the WT-CLP group, SIRT3 deficiency resulted in more increased expression of iNOS protein (Figures 2A and B). Moreover, we detected the expressions of antioxidant enzymes SOD and CAT in the kidneys. We found that the levels of antioxidant enzymes SOD and CAT in the WT-CLP group were significantly down-regulated compared with the WT-Sham group. Similar to this result, the levels of SOD and CAT in the kidneys of the KO-CLP group were also significantly reduced. However, in the KO-CLP group, SOD and CAT levels were reduced more (Figures 2C and D). These results indicated that loss of SIRT3 activated oxidative stress in septic mice.

***SIRT3 deficiency exacerbates mitochondrial dysfunction in CLP-induced AKI***

**Figure 3:**
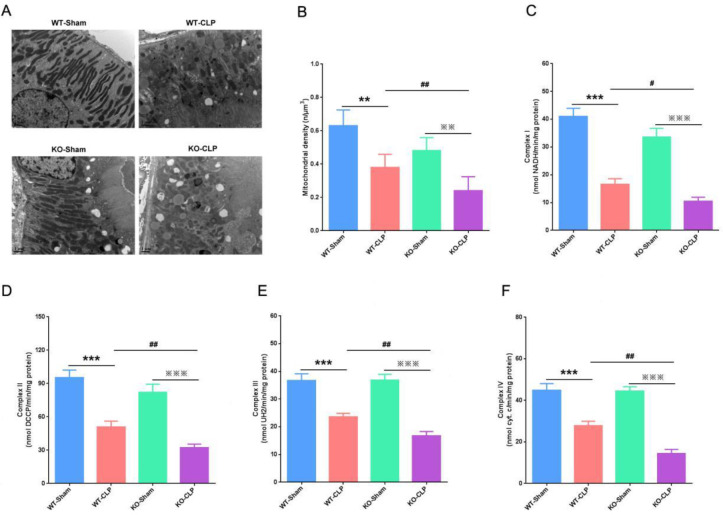
Protective effect of SIRT3 on mitochondrial function in CLP-induced AKI. (A) Effect of SIRT3 deficiency on the mitochondrial structure. (B) Quantitative determination of mean mitochondrial density (μm3) in proximal tubular cells. (C) Expression levels of complex I in the kidney. (D) Expression levels of complex II in the kidney. (E) Expression levels of complex III in the kidney. (F) Expression levels of complex IV in the kidney. SIRT3, Sirtuin3; CLP, cecal ligation and perforation; AKI, acute kidney injury. Compared with WT-Sham group, **P<0.01, ***P<0.001; compared with KO-Sham group,**P<0.01, ***P<0.001; compared with WT-CLP group, #*P*<0.05, ##*P*<0.01

To study the protective effect of SIRT3 on the mitochondrial function of KTEC in CLP-induced AKI, we observed the mitochondrial structure of KTEC by projection electron microscopy. We found that the mitochondrial structure of KTEC was complete and regular in both WT-Sham and KO-Sham groups. However, in the WT-CLP group, the mitochondria were swollen, irregular in structure, and the edges were blurred. In the KO-CLP group, the mitochondrial structure of KTEC was disordered and sparse, making it difficult to distinguish the edges (Figure 3A). Using morphometric analysis, we found that the average mitochondrial density of proximal tubular epithelial cells was significantly reduced in both WT-CLP and KO-CLP groups. Meanwhile, SIRT3 deficiency caused the average mitochondrial density to decrease more (Figure 3B). Also, we detected the levels of mitochondrial complex enzymes I/II/III/IV in the kidney. We found that mitochondrial complex enzymes I/II/III/IV were expressed at high levels in both WT-Sham and KO-Sham groups, and CLP caused these indicators to significantly reduce (Figures 3C-F). Furthermore, compared with the WT-CLP group, the mitochondrial complex enzymes I/II/III/IV of the KO-CLP group decreased more significantly (Figures 3C-F). The above results showed that the deficiency of SIRT3 exacerbated the mitochondrial structural disorder and dysfunction of AKI induced by CLP. 

***Effect of SIRT3 on apoptosis in CLP-induced AKI.***

**Figure 4:**
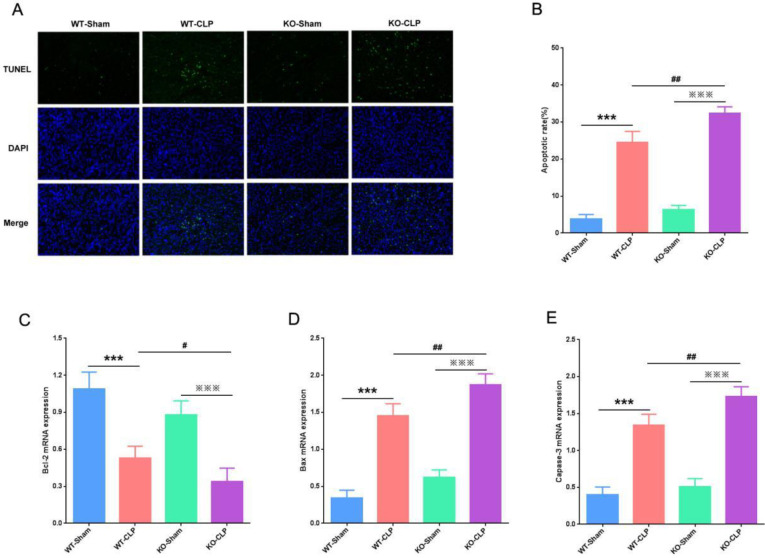
SIRT3 deficiency induces apoptosis of kidney tubular epithelial cells. (A) The apoptosis of kidney tissues detected by TUNEL assay. (B) Apoptotic rate of kidney in each group. (C) RT-qPCR detection of Bcl-2 mRNA in the kidney. (D) RT-qPCR detection of Bax mRNA in the kidney. (E) RT-qPCR detection of Caspase-3 mRNA in the kidney. SIRT3, Sirtuin3; TUNEL, terminal deoxynucleotidyl transferase-mediated dUTP-biotin nick end labeling assay. Compared with WT-Sham group, **P<0.01, ***P<0.001; compared with KO-Sham group, **P<0.01, ***P<0.001; compared with WT-CLP group, #*P*<0.05, ##*P*<0.01

In recent years, the role of apoptosis in septic AKI has been a hot research topic (13). To explore the effect of SIRT3 on the apoptosis of KTEC in CLP-induced AKI, we used the TUNEL method to detect the apoptosis of the kidney. We found that the number of apoptotic cells was very few in both WT-Sham and KO-Sham groups, But the number of apoptotic cells caused by CLP increased significantly. Besides, compared with the WT-CLP group, the apoptotic cells in the KO-CLP group increased more (Figures 4A and B). In addition, we used RT-qPCR to detect mRNA levels of Bcl-2, Bax, and Caspase-3 in the kidney. We found that the pro-apoptotic proteins Bax and Caspase-3 mRNA were low expression in both WT-Sham and KO-Sham groups, while the inhibitor of apoptosis protein Bcl-2 mRNA was expressed at a high level. However, CLP caused a significant increase in Bax and Caspase-3 mRNA, while it significantly decreased Bcl-2 mRNA. Compared with the WT-CLP group, Bax and Caspase-3 mRNA in the KO-CLP group increased significantly, while the Bcl-2 mRNA decreased (Figures 4C-E). Our results indicated that deficiency of SIRT3 induced the apoptosis of KTEC in septic mice. 

## Discussion

Sepsis is a common cause of AKI (1). In adults, sepsis accounts for 26%–50% of AKI in developed countries and accounts for 7%–10% of AKI related to primary kidney disease (3). Animal experiments showed that SIRT3 alleviates the pathological damage of the kidney and even prolongs the survival period (8). In the septic mice model induced by CLP, the generation of reactive oxygen species (ROS) is increased in kidney tissues of SIRT3 gene knockout mice, and SIRT3 reduces KTEC damage and apoptosis through the inflammatory response signaling pathway of NOD-like receptor family 3 inflammatory bodies, interleukin-1β and interleukin-12, thereby improving kidney function (11). 

In the present study, to investigate the protective effect of SIRT3 on CLP-induced AKI, we used immunohistochemistry and RT-qPCR to detect the expressions of SIRT3 protein and mRNA in the kidneys. We found that in the WT-CLP group, the SIRT3 protein and mRNA were expressed at lower levels in the kidneys, and the levels of Scr and pKIM-1 were significantly increased. However, Our study showed that SIRT3 knockout aggravated kidney pathological damage, these results confirmed the protective effects of SIRT3 on AKI induced by CLP. 

SOD and CAT are involved in the redox reaction in the human body, which can remove harmful metabolic substances produced after peroxidation and prevent lipid peroxidation (14). ROS are produced in the mitochondria of cells and are important biomarkers to judge the generation of oxygen free radicals and tissue damage (15). Under physiological conditions, ROS can regulate various physiological activities, such as cell homeostasis, cell division, and differentiation. However, under special circumstances, due to long-term ischemia, hypoxia, or toxic effects of drugs, the respiratory chain cannot normally obtain electrons. The respiratory chain is interrupted, and a large number of electrons accumulate, combining with the free oxygen ingested after reperfusion, which in turn generates ROS (16). 

Overexpression of SIRT3 inhibits the transcriptional activity of nuclear factor-κB, down-regulates the phosphorylation of extracellular regulatory protein kinases 1/2 and p38, and reduces the level of ROS. Therefore, there may be SIRT3-mediated resistance in the proximal tubular epithelial cells oxidation mechanism (17). To study the mechanism through which SIRT3 deficiency activates oxidative stress in CLP-induced AKI, we used immunohistochemistry to detect iNOS protein expression. Our results showed that SIRT3 knockout caused increased expression of iNOS protein, and reduced expressions of SOD and CAT in the kidneys. Our results indicated that loss of SIRT3 activated oxidative stress in sepsis and aggravated kidney damage.

AKI mainly refers to acute kidney tubular necrosis, and tubular cells are rich in mitochondria and have higher energy requirements. The proximal tubules are dependent on aerobic metabolic processes and are more likely to be in oxidative stress than the distal tubules (18). The mitochondrial changes of proximal tubules are an important sign of the development of kidney disease (19). To study the protective effect of SIRT3 on the mitochondrial function of the kidney in CLP-induced AKI, we observed the mitochondrial structure in the proximal tubular epithelial cells of the kidney by projection electron microscopy. Our results showed that SIRT3 knockout aggravated mitochondrial damage to the proximal tubules in the kidneys. Also, we detected the levels of mitochondrial complex enzymes I/II/III/IV in the kidneys. We found that SIRT3 deficiency led to a reduction in mitochondrial complex enzymes I/II/III/IV. These results indicated that loss of SIRT3 exacerbated mitochondrial structural disorders and dysfunction of AKI induced by CLP.

SIRT3 regulates the activity of enzyme complexes I/II/III/IV and mitochondrial ribosomal protein 10 in the respiratory chain, which can almost regulate the transcription of the entire respiratory chain. This indicated that protein acetylation regulation of SIRT3 may be the key factor in mitochondrial energy synthesis (20). The generation of energy is the core element of cell survival, and the reduction of ATP production is a strong signal of apoptosis (21). However, in the animal and cytology studies of AKI caused by sepsis, SIRT3 was not found to affect tubular epithelial cell apoptosis and apoptosis-related proteins. 

Therefore, to explore the effect of SIRT3 on the apoptosis of KTEC in septic AKI, we used the TUNEL method to detect the apoptotic cells of the kidney. Our results indicated that SIRT3 knockout resulted in a significantly increased number of apoptotic cells and that pro-apoptosis-related proteins Bax and Caspase-3 mRNA were significantly increased, and apoptosis-inhibiting protein Bcl-2 mRNA was significantly reduced. These results confirmed that lack of SIRT3 induced apoptosis of KTEC, thereby aggravating kidney injury.

## Conclusion

Our study indicates that SIRT3 deficiency promotes CLP-induced AKI via increasing oxidative stress, inducing mitochondrial dysfunction and apoptosis. There is no effective early treatment method for septic AKI, but SIRT3 as a mitochondrial protein is expected to become an effective treatment target.
